# Outcomes of concurrent chemoradiotherapy versus chemotherapy alone for advanced-stage unresectable intrahepatic cholangiocarcinoma

**DOI:** 10.1186/1748-717X-8-292

**Published:** 2013-12-21

**Authors:** Young-Il Kim, Joong-Won Park, Bo Hyun Kim, Sang Myung Woo, Tae Hyun Kim, Young Hwan Koh, Woo Jin Lee, Chang-Min Kim

**Affiliations:** 1 Center for Liver Cancer, National Cancer Center, 323 Ilsan-ro, Ilsan dong-gu, Goyang, Gyeonggi 411-769, South Korea

**Keywords:** Chemoradiotherapy, Cholangiocarcinoma, Intrahepatic, Unresectable

## Abstract

**Background:**

A standard treatment for unresectable advanced-stage intrahepatic cholangiocarcinoma (IHCC) has not yet been established. Although neoadjuvant concurrent chemoradiotherapy (CCRT) and liver transplantation are associated with long-term survival in select patients, the outcomes of CCRT for advanced-stage unresectable IHCC remain unclear. The aim of our study was to evaluate the outcomes of CCRT in patients with unresectable advanced-stage IHCC.

**Methods:**

We retrospectively reviewed the records of all patients with unresectable advanced stage (stage IVa or IVb) IHCC who were pathologically diagnosed and treated at National Cancer Center, Korea, from June 2001 to March 2012. Of the total of 92 patients, 25 (27.1%) received capecitabine plus cisplatin (XP) chemotherapy with external radiotherapy (RT) (XP-CCRT group) and 67 (72.8%) received XP chemotherapy alone (XP group). The clinical characteristics and outcomes of the 2 groups were compared.

**Results:**

The 92 patients comprised 72 male and 20 female patients, with a median age of 58 years (range 26–78 years). The baseline clinical characteristics of the 2 groups were similar. Patients in the XP-CCRT group received a mean 44.7 Gy of RT and a mean 5.6 cycles of XP chemotherapy, whereas patients in the XP group received a mean 4.0 cycles. The disease control rate was higher in the XP-CCRT group than in the XP group, but the difference was not statistically significant (56.0% vs. 41.5%, p = 0.217). Although neutropenia was significantly more frequent in the XP-CCRT than in the XP group (48% vs. 9%, p < 0.001), the rates of other toxicities and > grade 3 toxicities did not differ. At a median follow-up of 5.3 months, PFS (4.3 vs. 1.9 months, p = 0.001) and OS (9.3 vs. 6.2 months, p = 0.048) were significantly longer in the XP-CCRT than in the XP group.

**Conclusions:**

XP-CCRT was well tolerated and was associated with longer PFS and OS than XP chemotherapy alone in patients with unresectable advanced IHCC. Controlled randomized trials are required to determine whether XP-CCRT is a primary treatment option for patients with unresectable advanced IHCC.

## Background

Intrahepatic cholangiocarcinoma (IHCC) is a cancer arising from the intrahepatic bile duct, between the periphery of the liver and the second order bile duct. IHCC is the second most frequent type of primary liver cancer, after hepatocellular carcinoma [1]. The incidence of and mortality associated with IHCC have been increasing, but its prognosis remains poor because of a lack of effective treatment options [2-5]. Although surgical resection is potentially curative, more than half of these patients are at an advanced stage at the time of diagnosis and therefore have dismal prognosis. Resectability rates are generally quite low, but vary from 18%–70% [6]. The 5-year survival rate after surgery is modest at approximately 30% [7-9].

A large observational study reported that the median survival of untreated patients with advanced-stage, unresectable IHCC was 3 months [10]. To date, however, a treatment strategy for these patients has not yet been standardized. Palliative systemic chemotherapy has been found to prolong median survival compared with supportive care alone [11,12]. Neoadjuvant concurrent chemoradiotherapy (CCRT) followed by liver transplantation has been reported to be effective for select patients with early-stage unresectable perihilar cholangiocarcinoma [13,14] and locally advanced IHCC [15,16]. Although CCRT has been also found to improve survival in patients with advanced-stage unresectable perihilar or extrahepatic cholangiocarcinoma [17,18], it is not clear if CCRT also prolongs survival in patients with advanced-stage unresectable IHCC. We have therefore retrospectively compared outcomes, including response rate, progression-free survival (PFS), overall survival (OS), and safety, in patients with advanced-stage unresectable IHCC, including those with distant metastases, who were treated with CCRT or systemic chemotherapy alone.

## Methods

### Patients

**Figure 1:**
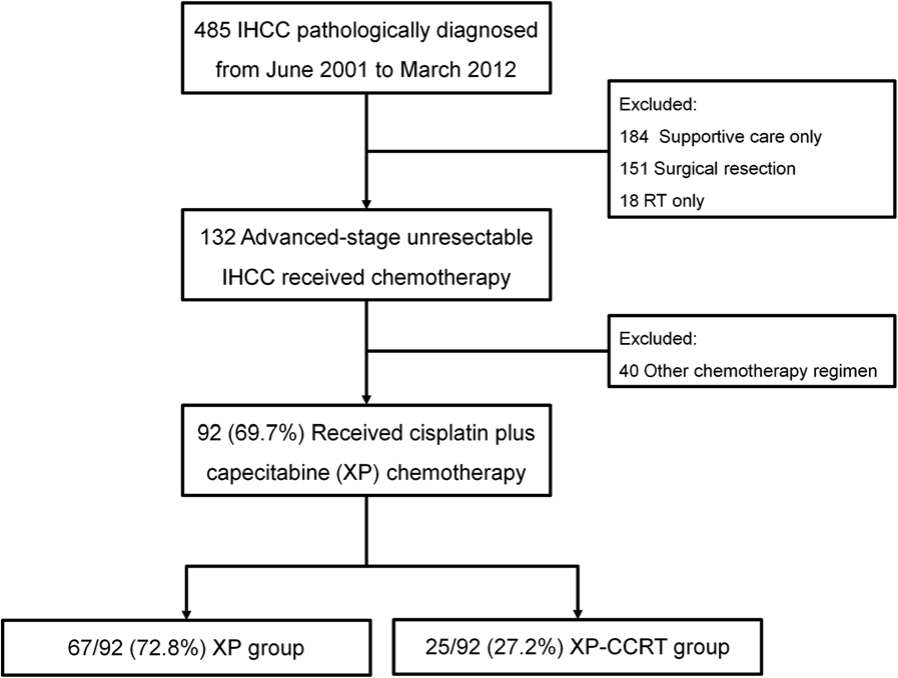
Patient flow chart.

Hospital records of 485 patients who were pathologically diagnosed, by ultrasonography-guided percutaneous liver biopsy, with IHCC at the National Cancer Center, Korea, from June 2001 to March 2012 were retrospectively reviewed. IHCC resectability was determined by clinical and radiographic findings, with extensive bilobular invasion, major vessel involvement, and extrahepatic metastases considered markers of unresectable disease. Of the 132 patients with advanced-stage unresectable IHCC who received palliative systemic chemotherapy, 92 (69.7%) were treated with capecitabine plus cisplatin (XP) and were retrospectively analyzed. All patients were found to have stage IVa (46.7%) or IVb (53.3%) disease according to the seventh edition of the American Joint Committee on Cancer-TNM staging system [19]. Of the 92 included patients, 25 (27.2%) received XP chemotherapy with concurrent radiotherapy (XP-CCRT group) and 67 (72.8%) received XP chemotherapy alone (XP group). Patients who underwent surgical resection, those who were treated with other chemotherapy regimens, and those who received supportive care alone were excluded (Figure 1). The present study was approved by the Institutional Review Board of the National Cancer Center, Korea (NCCNCS-12-686), and was conducted in accordance with the guidelines of the International Conference on Harmonization/Good Clinical Practice and the principles of the Declaration of Helsinki.

### Chemotherapy

Each patient received 1000 mg/m^2^ oral capecitabine twice daily for the first 14 days of each 21-day cycle, followed by a 7-day rest period, together with 30 mg/m^2^ intravenous cisplatin for 1 hour with standard hydration on days 1 and 8 of each cycle [20]. Patients were continued on XP chemotherapy until progressive disease or the development of severe toxicity.

### Radiotherapy

Among included patients, 25 patients underwent concurrent RT. Eligible criteria for RT included: 1) radiographically assessable disease, 2) adequate laboratory findings including common blood cell counts (white blood cell ≥ 2,000/mm^3^, hemoglobin ≥ 7.5 g/dL, platelet ≥ 100,000/mm^3^), total bilirubin ≤ 3.0 mg/dL, serum creatinine ≤ 1.5 mg/dL), 3) adequate oral intake of 1,500 calories/day. Meanwhile, exclusion criteria for RT were as follows: 1) previous history of RT adjacent to the planned RT field, 2) multifocal intrahepatic lesions which did not covered by planned RT field.

Planning of external beam radiotherapy (RT) was based on radiographic findings from abdominal computed tomography (CT) and/or magnetic resonance imaging. The gross tumor volume was defined by these planning imaging studies. Then, the clinical target volume included the gross tumor volume and the volumes of adjacent lymph nodes (LNs) in the porta hepatis, celiac axis, and pancreaticoduodenal ligament, regardless of the presence of extrahepatic metastases. The clinical target volume and gross tumor volume plus a 5–10 mm margin were included in the initial and boot planning target volume. Of the 25 patients in this group, 18 (72.0%) were treated with 3-dimensional conformal RT using 2–4 ports, 5 (20.0%) received intensity-modulated RT using 2–5 ports, and 2 (8.0%) received proton beam RT using 2 ports. All these patients underwent a CT simulation in the treatment position. In this treatment planning, the planning target volume would be encompassed by a 90% iso-dose volume of the prescribed dose.

Concurrent RT was applied in single fractions of 2.0–3.0 Gy once a day and 5 times a week, with a mean total RT dose of 44.7 Gy (range 25.0–60.0 Gy). Although usual target doses were between 37.5 Gy and 50.0 Gy, several fractions of booster RT were performed in some well-tolerate patients with limit dose of 60.0 Gy.

### Follow-up and assessment of tumor response and toxicity

Patients were evaluated by physical examination and assessments of complete blood counts and blood chemistry after each cycle. Abdominal CT was performed after every 2 cycles of chemotherapy to measure tumor response, which was classified according to the Response Evaluation Criteria in Solid Tumor (RECIST) guidelines [21]. PFS was defined as the time from diagnosis to the development of progressive disease (PD) or death whichever came first. Toxicity was monitored according to the Common Terminology Criteria for Adverse Events (CTCAE) v3.0.

### Statistical analysis

Comparisons between 2 groups were evaluated using Student’s *t* tests for continuous variables or chi-square tests for categorical variables, as appropriate. The follow-up period of each patient was defined from the time of diagnosis to the time of last visit or discharge from hospital. Among 92 patients included in this study, 91 were dead, and the death dates of patients who did not die in hospital were obtained from the claim database of Korean National Health Insurance Corporation after permission of data access. Estimated OS and radiographic PFS were calculated from the time of diagnosis using the Kaplan-Meier method and analyzed using log-rank tests. All statistical analyses were performed using STATA 12.1 for Windows (Stata Corp, College Station, TX, USA). A p value of <0.05 was considered statistically significant.

## Results

### Patient characteristics

Table 1 shows the baseline clinical characteristics of the 92 enrolled patients. Of these patients, 72 (78.3%) were male and 20 (21.7%) were female; their median age was 58 years (range 26–78 years). Eighty-seven patients (94.6%) had good performance status (Eastern Cooperative Oncology Group [ECOG] scale 0 or 1). The mean maximum tumor size was 7.3 cm, and the mean serum concentrations of carbohydrate antigen (CA) 19–9 and carcinoembryonic antigen (CEA) were 2675.4 U/mL and 107.7 ng/mL, respectively. Evaluation using the morphologic classification system proposed by the Liver Cancer Study Group of Japan [22] indicated that 79 (85.9%) patients had mass-forming type and 13 (14.1%) had periductal infiltrative type IHCC. In addition, 76 patients (82.6%) had LN metastasis and 49 (53.3%) had extrahepatic metastasis. Patients in XP-CCRT group had significantly higher rate of single intrahepatic lesion than those in XP group (72.0% vs. 41.3%, p = 0.007), however, other baseline clinical characteristics of the XP-CCRT and XP groups did not differ significantly.

**Table 1:** Baseline clinical characteristics of the 92 enrolled patients

	**CCRT (n = 25)**	**Chemotherapy (n = 67)**
Median age at diagnosis, years (range)	56 (32 – 75)	58 (26 – 78)
Sex, n (%)		
Male	19 (76.0)	53 (79.1)
Female	6 (24.0)	14 (20.9)
ECOG, n (%)		
0	10 (40.0)	39 (58.2)
1	14 (56.0)	24 (35.8)
2	1 (4.0)	4 (6.0)
Tumor stage, n (%)		
Stage IVa	14 (56.0)	29 (43.3)
Stage IVb	11 (44.0)	38 (56.7)
Tumor characteristics		
Maximum diameter, cm	7.6 ± 3.9	7.4 ± 3.1
Location of intrahepatic lesions, n (%)		
Unifocal	18 (72.0)	27 (40.7)
Multifocal	7 (28.0)	40 (59.7)
Radiographic gross type, n (%)		
Mass-forming	22 (88.0)	57 (85.1)
Periductal infiltrative	3 (12.0)	10 (14.9)
LN metastasis, n (%)	21 (84.0)	55 (82.1)
Extrahepatic metastasis, n (%)	11 (44.0)	38 (56.7)
CA 19–9, U/mL	1684.7 ± 4881.2	3121.7 ± 9622.1
CEA, ng/mL	26.5 ± 53.9	190.3 ± 764.0

Data expressed as number (%) or mean ± standard deviation.

### Tumor response and outcomes

Patients in the XP-CCRT group received more cycles of chemotherapy than those in the XP group (5.6 vs. 4.0 cycles, p = 0.082). Of the 92 patients, tumor response could not be assessed in 2 patients because of a lack of follow-up CT data. Of the 90 patients evaluable for tumor response, 4 (4.4%) had a partial response (PR) and 37 (41.1%) had stable disease (SD). Disease control rate (DCR), which accounted for patients with complete response (CR), PR, and SD, was higher in the XP-CCRT than in the XP group, although the difference was not statistically significant (56.0% vs. 41.5%, p = 0.217) (Table 2).

**Table 2:** Tumor response and outcomes of the 92 patients

	**CCRT (n = 25)**	**Chemotherapy (n = 67)**	**p-value**
Capecitabine plus cisplatin chemotherapy, total cycles (mean ± SD)	5.6 ± 5.8	4.0 ± 3.2	0.082
Tumor response (n = 90), n (%)			0.365
CR	0 (0)	0 (0)	
PR	1 (4.0)	3 (4.6)	
SD	13 (52.0)	24 (36.9)	
PD	11 (44.0)	38 (58.5)	
Disease-control rate (CR + PR + SD)	14 (56.0)	27 (41.5)	0.217
Median FU duration, months (IQR)	7.4 (4.1 – 15.1)	4.1 (2.3 – 8.3)	0.044
Median PFS, months (95% CI)	4.3 (3.3 – 5.4)	1.9 (1.3 – 2.4)	0.001*
Median OS, months (95% CI)	9.3 (7.6 – 11.0)	6.2 (4.1 – 8.2)	0.162*

*By log-rank test.

**Figure 2:**
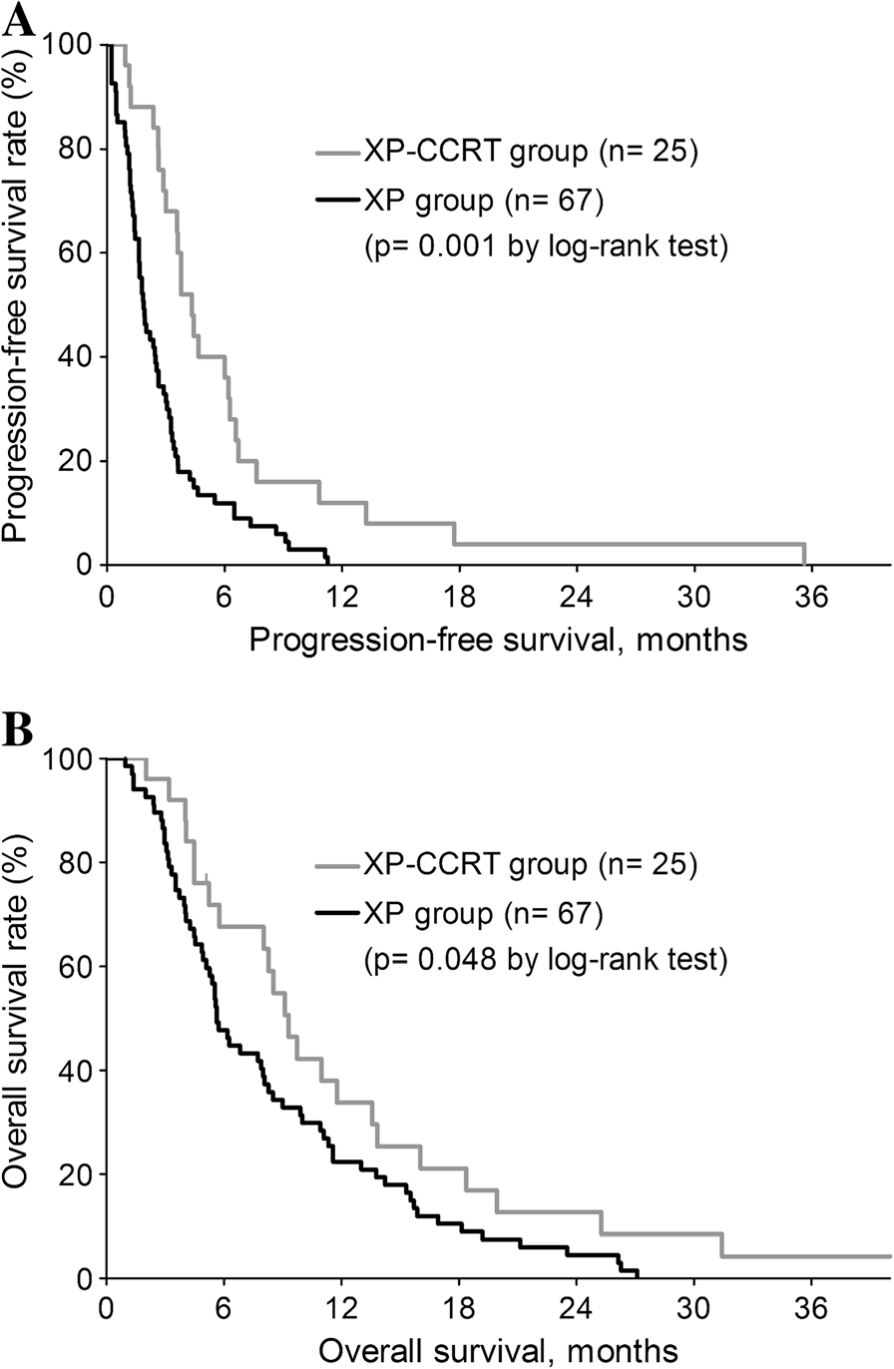
Kaplan-Meier estimates of progression-free survival (A) and overall survival (B) in the XP-CCRT and XP groups.

The median follow-up duration of the 92 patients was 5.3 months (interquartile range [IQR], 3.0–9.5 months). The median PFS for all patients was 2.6 months (95% confidential interval [CI] 2.0–3.2 months), and the median OS was 7.9 months (95% CI 5.8–10.0 months). Median PFS (4.3 vs. 1.9 months, p = 0.001) and OS (9.3 vs. 6.2 months, p = 0.048) were significantly longer in the XP-CCRT than in the XP group (Figure 2, Table 2), but the 1-year survival rates did not differ significantly (30.4% vs. 22.4%, p = 0.438).

### Toxicity

The rates of hematologic and non-hematologic toxicities are listed in Table 3. Neutropenia and hand-foot syndrome were more frequent in the XP-CCRT than in the XP group. However, the frequency of grade 3 and 4 toxicities did not differ between the groups. In the XP-CCRT group, 3 patients (12.0%) had grade 3 neutropenia without fever, 5 (20.0%) had grade 3 thrombocytopenia, and 1 (4.0%) had grade 4 hand-foot syndrome. Treatment was well tolerated in both treatment groups and there were no treatment-related deaths.

**Table 3:** Toxicities associated with treatment

	**CCRT (n = 25)**	**Chemotherapy (n = 67)**	**p-value**
Hematologic toxicity			
Neutropenia	12 (48.0)	6 (9.0)	< 0.001
Thrombocytopenia	17 (68.0)	36 (53.7)	0.218
Anemia	13 (52.0)	25 (37.3)	0.203
Non-hematologic toxicity			
Anorexia	16 (64.0)	28 (41.8)	0.058
Nausea	8 (32.0)	16 (23.9)	0.430
Vomiting	3 (12.0)	5 (7.5)	0.678
Asthenia	6 (24.0)	28 (41.8)	0.116
Dyspnea	17 (68.0)	36 (53.7)	0.218
Peripheral neuropathy	3 (12.0)	3 (4.5)	0.339
Hand-foot syndrome	6 (24.0)	3 (4.5)	0.011
>Grade 3 toxicity	6 (24.0)	7 (10.4)	0.174

## Discussion

Patients with unresectable IHCC may be eligible for local-regional therapy, systemic chemotherapy, and best supportive care. Local-regional therapies for these patients include RT, transarterial chemoembolization, and radiofrequency ablation, but few studies to date have evaluated their efficacy in patients with unresectable IHCC. To our knowledge, this is the first study to assess the outcomes of CCRT in patients with stage IVa/IVb IHCC. We found that the combination of XP chemotherapy and external beam RT was well tolerated and resulted in prolonged PFS and OS compared with XP alone. CCRT outcomes have been reported in patients with extrahepatic cholangiocarcinoma [17,18,23,24], and CCRT did not significantly enhance benefits, compared with RT alone, in patients with unresectable extrahepatic biliary cancer [25]. Prospective randomized trials are therefore needed to confirm the benefits of CCRT in patients with advanced-stage unresectable biliary tract cancer.

A recent retrospective study showed that external beam RT prolonged survival and relieved the symptoms of jaundice in patients with early-stage but unresectable IHCC, with a median OS of 9.5 months and a 1-year OS rate of 38.5% [26]. However, CCRT may be more beneficial than loco-regional RT alone for patients with advanced-stage unresectable IHCC, including those with distant metastases. The CCRT group in our study, which included 11 patients (44%) with extrahepatic metastases, had better outcomes than the group receiving XP alone. Because of the small number of patients, however, we could not analyze the prognostic factors, such as distant metastasis, associated with OS and PFS.

Although systemic chemotherapy has been reported superior to best supportive care [11,12], a standard chemotherapy regimen has not yet been established in patients with advanced, unresectable IHCC, because of the lack of randomized, prospective trials [27]. A recent phase III randomized controlled trial showed that the combination of gemcitabine and cisplatin (GP chemotherapy) was highly effective in patients with locally advanced or metastatic biliary tract cancer, including in patients with recurrent disease after resection [28]. Because on a few patients in our center received GP chemotherapy, we analyzed patients treated with XP chemotherapy. In phase II trials, XP chemotherapy was shown to be an active and well-tolerated first-line regimen in patients with advanced biliary tract cancer [29,30].

XP has also shown good radio-sensitizing activity in patients with gastrointestinal malignancies [31]. Our findings indicated that CCRT may be better option for patients with advanced-stage unresectable IHCC than systemic chemotherapy alone. The better outcomes observed with XP-CCRT may have been because of the synergistic effects of RT and systemic chemotherapy. Moreover, except for neutropenia, the frequencies of treatment-related toxicity and >3 grade toxicity (24% vs. 10.4%, p = 0.174) were not significantly higher in the XP-CCRT than in the XP group, despite the former group receiving more cycles of chemotherapy as well as RT. These findings indicate that XP-CCRT is safe for patients with advanced-stage, unresectable IHCC.

This study had several limitations, including the small number of patients and its retrospective design. Thus, selection bias is inevitable. Moreover, the RT method and total dose differed among patients in the XP-CCRT group. Nevertheless, the significant improvements in PFS and OS observed with CCRT suggest the need for randomized prospective clinical trials comparing the different chemotherapy regimens (e.g., GP vs. XP) in combination with RT.

## Conclusion

XP-CCRT was well tolerated and resulted in prolonged PFS and OS compared with XP chemotherapy alone in patients with unresectable advanced IHCC. Controlled randomized trials are required to determine whether XP-CCRT should be a primary treatment option for these patients.

## Abbreviations

CA: Carbohydrate antigen; CCRT: Concurrent chemoradiotherapy; CEA: Carcinoembryonic antigen; CI: Confidential interval; CT: Computed tomography; ECOG: Eastern cooperative oncology group; IHCC: Intrahepatic cholangiocarcinoma; IQR: Interquartile range; LN: Lymph node; OS: Overall survival; PFS: Progression-free survival; PR: Partial response; RT: Radiotherapy; SD: Stable disease; XP: Capecitabine plus cisplatin

## Competing interests

The authors declared that they have no competing interests.

## Authors’ contributions

JWP designed the study concept. YIK and JWP carried out the data collection and drafted the manuscript. YIK, JWP, BHK, SMW, WJL participated in the data collection. YHK, WJL and CMK provided comments, critique and suggestions for its improvement. THK carried out radiotherapy planning and provided comments, critique and suggestions for its improvement. All authors read and approved the final manuscript.
